# PEI-Coated Fe_3_O_4_ Nanoparticles Enable Efficient Delivery of Therapeutic siRNA Targeting REST into Glioblastoma Cells

**DOI:** 10.3390/ijms19082230

**Published:** 2018-07-31

**Authors:** Rui Wang, Volkan Degirmenci, Hongchuan Xin, Ying Li, Liping Wang, Jiayu Chen, Xiaoyu Hu, Dianbao Zhang

**Affiliations:** 1 Department of Stem Cells and Regenerative Medicine, Key Laboratory of Cell Biology, Ministry of Public Health of China, and Key Laboratory of Medical Cell Biology, Ministry of Education of China, China Medical University, Shenyang 110122, China; rwang@cmu.edu.cn (R.W.); liying@cmu.edu.cn (Y.L.); lpwang@cmu.edu.cn (L.W.); chenjiayu@cmu.edu.cn (J.C.); 2 School of Engineering, University of Warwick, Coventry CV4 7AL, UK; v.degirmenci@warwick.ac.uk; 3 Qingdao Institute of Bioenergy and Bioprocess Technology, Chinese Academy of Sciences, Qingdao 266101, China; xinhc@qibebt.ac.cn; 4 College of Basic Medical Science, China Medical University, Shenyang 110122, China; xyhu@cmu.edu.cn

**Keywords:** nanoparticles, iron oxide, siRNA delivery, REST/NRSF, glioblastoma, gene therapy

## Abstract

Glioblastomas (GBM) are the most frequent brain tumors lacking efficient treatment. The increasingly elucidated gene targets make siRNA-based gene therapy a promising anticancer approach, while an efficient delivery system is urgently needed. Here, polyethyleneimine (PEI)-coated Fe_3_O_4_ nanoparticles (NPs) have been developed and applied for siRNA delivery into GBM cells to silence repressor element 1-silencing transcription factor (REST). The prepared PEI-coated Fe_3_O_4_ NPs were characterized as magnetic nanoparticles with a positive charge, by transmission electronic microscopy, dynamic light-scattering analysis and a magnetometer. By gel retardation assay, the nanoparticles were found to form complexes with siRNA and the interaction proportion of NP to siRNA was 2.8:1. The cellular uptake of NP/siRNA complexes was verified by prussian blue staining, fluorescence labeling and flow cytometry in U-87 and U-251 GBM cells. Furthermore, the REST silencing examined by realtime polymerase chain reaction (PCR) and Western blotting presented significant reduction of REST in transcription and translation levels. Upon the treatment of NP/siRNA targeting REST, the GBM cell viabilities were inhibited and the migration capacities were repressed remarkably, analyzed by cell counting kit-8 and transwell assay separately. In this study, we demonstrated the PEI-coated Fe_3_O_4_ nanoparticle as a vehicle for therapeutic siRNA delivery, at an appropriate NP/siRNA weight ratio for REST silencing in GBM cells, inhibiting cell proliferation and migration efficiently. These might represent a novel potential treatment strategy for GBM.

## 1. Introduction

Glioblastomas (GBM, World Health Organization (WHO) grade IV) are the most frequent and invasive malignant primary tumors of the central nervous system [1,2]. Currently, treatments available for GBM patients are limited to surgery, non-specific chemotherapy and radiotherapy [3]. Despite improvements in these conventional therapies, the median survival of patients with GBM is only approximately 15 months and only 3%–5% of the patients survive more than 3 years [4]. Thus, novel treatment strategies for GBM are needed urgently.

Gene therapy is the delivery of therapeutic nucleic acid (including DNA, antisense oligonucleotides and short interfering RNA (siRNA), etc.) into a patient’s cells as a drug to treat diseases [5]. Specifically designed double-stranded siRNA could induce the specific degradation of homologous messenger RNA, leading to gene silencing [6]. siRNA has been widely used as a research tool for studying gene functions and investigated as a novel therapeutic strategy against various disorders including cancers [7]. Since increasing gene targets have been elucidated and any gene can be targeted by siRNA, siRNA-based gene therapy is considered a promising new anticancer approach.

The siRNA is easy to degrade by widely-existing Ribonuclease (RNase), and it possesses the same negative charge as the cell membrane. Accordingly, siRNA cannot be readily taken up by cells and efficient delivery vehicles for siRNA are critical for the delivery of therapeutic siRNA. During the past decade, several types of nanoparticle (NP) delivery systems have been developed, including inorganic NPs, lipid-based nanocarriers, polymeric NPs, dendrimers, solid lipid NPs, and carbon NPs [8,9]. For GBM, siRNA targeting MGMT (O_6_-methylguanine methyltransferase, a key factor in brain tumor resistance to TMZ) delivered by iron oxide NPs conjugated with chlorotoxin could enhance temozolomide (TMZ) toxicity [10]. Polyethyleneimine (PEI)-entrapped gold NP modified with an arginine-glycine-aspartic peptide was capable of delivering siRNA targeting Bcl-2 to GBM cells to increase cell apoptosis [11]. In our previous studies, a type of cationic PEI-coated Fe_3_O_4_ NPs were proved to be effective in siRNA protection and siRNA delivery into mesenchymal stem cells [12]. The capacity of PEI-coated Fe_3_O_4_ NPs to deliver siRNA into GBM cells for gene silencing was unknown.

Repressor element 1-silencing transcription factor (REST, also named NRSF and XBR) is a zinc-finger transcription factor belong to krüppel family, it binds to a specific DNA motif (repressor element 1) within regulatory regions to repress gene transcription [13]. We demonstrated in our previous study that REST could work as a master regulator which maintains cell proliferation and migration in GBM cells, which might represent a potential target for GBM therapy [14]. In the present study, we aimed to investigate the PEI-coated Fe_3_O_4_ NPs for the delivery of therapeutic siRNA targeting REST into GBM cells, providing a novel treatment strategy for GBM.

## 2. Results

### 2.1. Characterization of Polyethyleneimine (PEI)-Coated Fe_3_O_4_ Nanoparticles (NPs)

**Figure 1:**
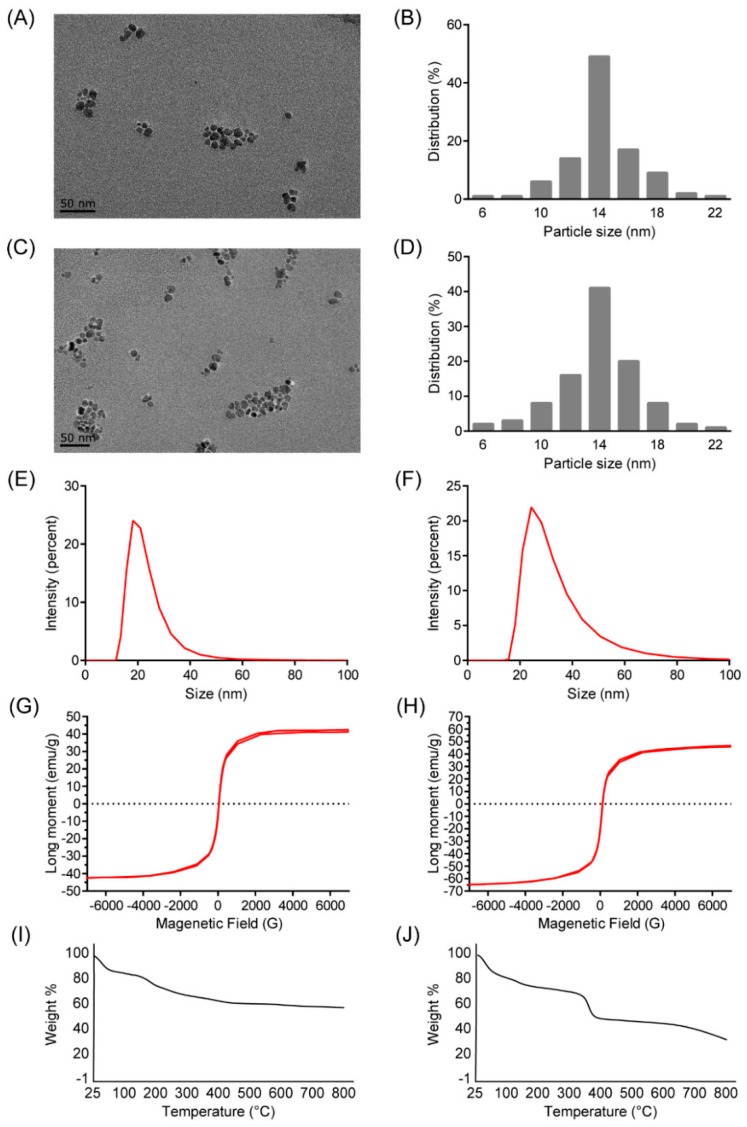
Characterization of the polyethyleneimine (PEI)-coated Fe_3_O_4_ NPs. (**A**) The transmission electron microscope (TEM) image of the Fe_3_O_4_ NPs without PEI. Scale bar indicates 50 nm; (**B**) the particle size distribution of the Fe_3_O_4_ NPs without PEI, from the analysis of TEM images; (**C**) the TEM image of the PEI-coated Fe_3_O_4_ NPs. Scale bar indicates 50 nm; (**D**) the particle size distribution of the PEI-coated Fe_3_O_4_ NPs, from the analysis of TEM images; (**E**) the hydrodynamic size of the Fe_3_O_4_ NPs without PEI, analyzed by dynamic light scattering (DLS). (**F**) the hydrodynamic size of the PEI-coated Fe_3_O_4_ NPs, analyzed by DLS; (**G**) The magnetization curve of the Fe_3_O_4_ NPs without PEI was analyzed by magnetization curve obtained by vibrating sample magnetometer (VSM) at room temperature; (**H**) The magnetization curve of the PEI-coated Fe_3_O_4_ NPs was analyzed by magnetization curve obtained by vibrating sample magnetometer (VSM) at room temperature; (**I**) the thermogravimetric analysis curve of the Fe_3_O_4_ NPs without PEI; (**J**) the thermogravimetric analysis curve of the PEI-coated Fe_3_O_4_ NPs.

The prepared PEI-coated Fe_3_O_4_ NPs were water soluble and characterized by transmission electron microscope (TEM), dynamic light scattering (DLS) and magnetometer. In the TEM image of the PEI-coated Fe_3_O_4_ NPs, the morphology of the NPs was compact and close to spherical, with a diameter of about 14 nm, which was the same as Fe_3_O_4_ NPs without PEI coating (Figure 1A–D). However, it was difficult to observe the PEI coating on the NPs directly by TEM, due to the transparence of PEI under TEM. Subsequently, the PEI-coated Fe_3_O_4_ NPs were analyzed by DLS, it showed the hydrodynamic size of 18.21 nm and the zeta potential of 25.7 mV, indicating the small hydrodynamic size and positive net charge of the NPs (Figure 1F). While the results of NPs without PEI coating showed the hydrodynamic size of 21.83 nm and the zeta potential of −41.4 mV (Figure 1E). The magnetization curve of the Fe_3_O_4_ NPs was given in Figure 1G,H. The curve does not show any hysteresis loop which is characteristic for superparamagnetic Fe3O4 NPs. The saturation magnetization is obtained as 47.0 emu/g for the NPs and 42.5 emu/g for the NPs without PEI. In addition, the binding efficiency of PEI on magnetic nanoparticles (MNPs) was analyzed by thermogravimetric analysis, and it was found that the binding of PEI on MNP was 18% (Figure 1I,J).

### 2.2. Interaction between the NP and siRNA

**Figure 2:**
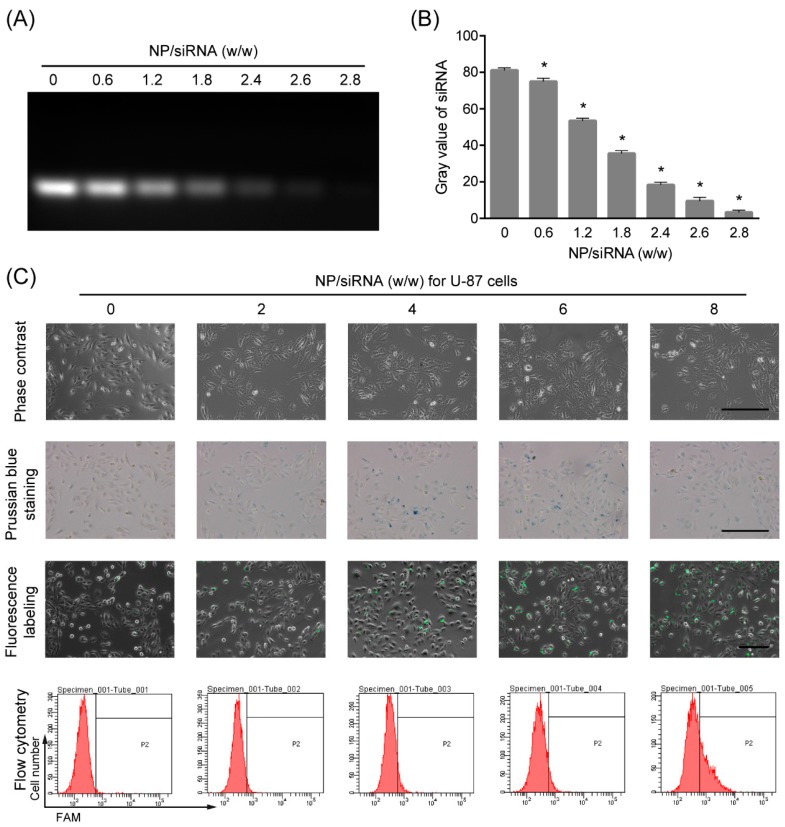
The cellular uptake of the NP/siRNA complexes in U-87 cells. (**A**) The interaction of the PEI-coated Fe_3_O_4_ NPs with siRNA at different NP/siRNA weight ratios, analyzed by gel retardation assay; (**B**) the gray value analysis for the results of gel retardation assay. * *p* < 0.05 compared with the 0 group; (**C**) the cellular uptake of the NP/siRNA complexes in U-87 cells was verified by prussian blue staining, fluorescence labeling and flow cytometry, P2 presented positive proportions. Bar indicates 100 μm.

For achieving siRNA delivery, the delivery reagents are required to form complexes with siRNA in vitro. Here, the capacity of the PEI-coated Fe_3_O_4_ NPs to interact with siRNA was examined by gel retardation assay. As shown in Figure 2A,B, the observed siRNA was reduced in a ratio-dependent manner when incubated with the PEI-coated Fe_3_O_4_ NPs and almost completely retarded at a weight ratio of 2.8:1. It is suggested that the PEI-coated Fe_3_O_4_ NPs could form complexes with siRNA to block siRNA migration in the agarose gel electrophoresis, and the interaction proportion of NP to siRNA was 2.8:1. In addition, the size of the siRNA-loaded NPs has been determined to be 43.56 nm by DLS when the ratio of NPs/siRNA was 4, which proved the stability of the NP/siRNA complexes.

### 2.3. Cellular Uptake of the NP/siRNA Complexes

**Figure 3:**
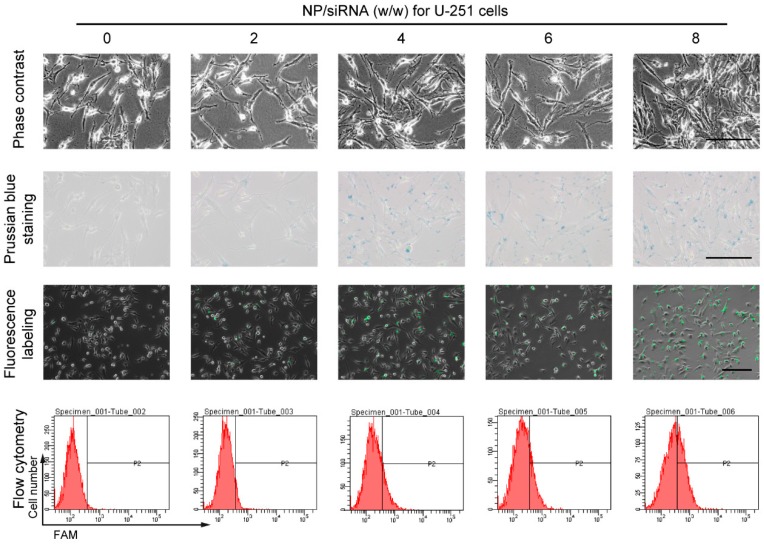
The cellular uptake of the NP/siRNA complexes in U-251 cells. The cellular uptake of the NP/siRNA complexes in U-251 cells was verified by prussian blue staining, fluorescence labeling and flow cytometry, P2 presented positive proportions. Bar indicates 100 μm.

To verify the siRNA delivery into GBM cells by the PEI-coated Fe_3_O_4_ NPs, two human GBM cell lines U-87 and U251 cells were used. The cells were incubated with NP/siRNA complexes at the ratio of 0, 2, 4, 6 and 8 for 6 h, respectively. The cellular uptakes of the NPs stained with prussian blue are shown in Figure 2C and Figure 3. It was observed that almost all the cells were stained blue and darker when the NP/siRNA ratio was 4 or 6. Furthermore, 5-Carboxyfluorescein (FAM)-conjunct siRNA was used to analyze the cellular uptake of siRNA delivered by the NPs. Under the fluorescence microscope, the fluorescence labeling of siRNA was observed, indicating the cellular uptake of the siRNA. The labeling efficiencies were detected using flow cytometry and it showed a NP/siRNA ratio-dependent behavior. Nevertheless, the efficiencies detected by flow cytometry were lower than the results of prussian blue staining and fluorescence labeling. This could be due to the detection sensitivity and the quenching of fluorescein by NP. These results indicated efficient delivery of siRNA in to GBM cells by the PEI-coated Fe_3_O_4_ NPs.

### 2.4. REST (Repressor Element 1-Silencing Transcription Factor) Silencing Mediated by NP/siRNA Complexes

**Figure 4:**
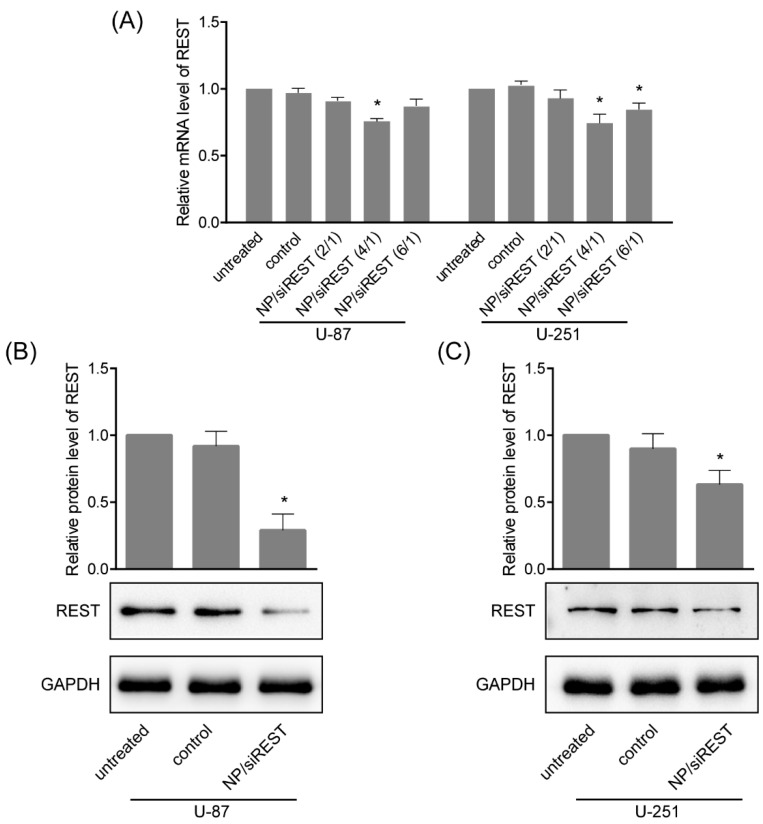
Repressor element 1-silencing transcription factor (REST) silencing mediated by NP/siRNA complexes in GBM cells. (**A**) The mRNA levels of REST in U-87 and U-251 cells incubated with NP/siRNA complexes (at the ratio of 4 h for 24 h) targeting REST, assayed by real-time polymerase chain reaction (PCR); (**B**) The protein levels in U-87 cells treated with NP/siRNA complexes targeting REST were detected by Western blotting; (**C**) The protein levels in U-251 cells treated with NP/siRNA complexes targeting REST were detected by western blotting. * *p* < 0.05 compared with the control group.

To verify the gene silencing mediated by NP/siRNA complexes in GBM cells, the cells were incubated with NP/siRNA complexes at the ratio of 4 for 24 h, real-time polymerase chain reaction (PCR) and Western blotting were carried out. As shown in Figure 4A, the mRNA levels of REST in U-87 and U-251 cells treated with NP/siRNA targeting REST was significantly reduced as compared to control experiments. Consistent with the trend of realtime PCR results, Western blotting showed stronger REST knockdown in U-87 and U-251 cells (Figure 4B,C), indicating that REST was silenced by NP/siRNA complexes mainly in transcription and translation levels.

### 2.5. Anti-Tumor Activity of NP/siRNA Complexes

**Figure 5:**
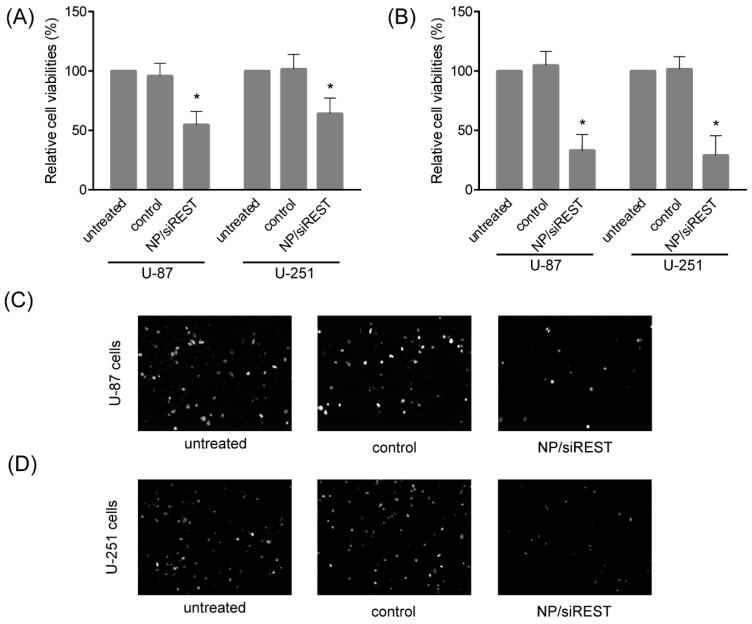
The GBM cell viability and migration were inhibited by NP/siRNA complexes. (**A**) The cell viabilities of U-87 and U-251 cells treated with NP/siRNA targeting REST for 24 h were analyzed by CCK-8 assay; (**B**) The cell viabilities of U-87 and U-251 cells treated with NP/siRNA targeting REST for 48 h were analyzed by CCK-8 assay; (**C**,**D**) The cell migration capacities of U-87 and U-251 cells treated with NP/siRNA targeting REST, analyzed using transwell assay. Bar indicates 100 μm. * *p* < 0.05 compared with the control group.

Cell viability and migration are crucial to GBM development and metastasis. The anti-tumor activity of REST-silencing mediated by the PEI-coated Fe_3_O_4_ NPs was determined using CCK-8 assay and transwell assay in U-87 and U-251 GBM cells. In the cell viability assay, the concentration of the NP/siRNA was 200 ng/50 ng. In Figure 5A,B, the results of the CCK-8 assay presented significant reduction of the cell viabilities upon siRNA against REST delivery by the PEI-coated Fe_3_O_4_ NPs, both in U-87 and U-251 cells. Moreover, the cell migration capacities of U-87 and U-251 cells were significantly inhibited by the NP/siRNA complexes targeting REST (Figure 5C,D). These data have proved the PEI-coated Fe_3_O_4_ NPs as a novel delivery system for siRNA into GBM cells, and the combination with siRNA for REST provided a novel promising potential strategy for GBM treatment.

## 3. Discussion

In recent years, studies on the molecular mechanisms of GBM occurrence, development and drug resistance have provided a lot of potential therapeutic targets, but they have not significantly promoted the progress of clinical treatment. The siRNA-based approaches make it possible for any abnormal highly expressed genes to be silenced, including undruggable genes. Nanomaterials have opened novel opportunities for the delivery of therapeutic molecules which cannot be readily taken up, such as siRNA [8]. The siRNA is a double-stranded RNA with typically 21–23 nucleotides having 3′ two-nucleotide overhangs [6]. Its negative charge allows the use of the cationic carriers including lipids, dendrimers, natural polymers and PEI [15]. Recently, lipopolymetric NP, PEI-functionalized gold nanorods and PEI NP have been reported in the NP/siRNA-based therapy strategies for GBM [16,17,18,19]. In a similar approach, we managed to produce PEI-coated Fe_3_O_4_ NP/siRNA complexes for its targeted delivery. The zeta potential of the PEI-coated Fe_3_O_4_ NP was measured as +25.7 mV and the net charge was reduced after forming complexes with negatively charged siRNA,. This could still facilitate the approaching of NP to a negatively charged cell membrane to promote NP/siRNA cellular uptake. The excessive siRNA could neutralize the net charge of the NP/siRNA complexes and reduce the cellular uptake, while excessive NP exceeded the cellular uptake ability and depressed the siRNA delivery. Therefore, the balance between the efficiency of NP cellular uptake and the efficiency of siRNA delivery has been considered in the optimization of delivery strategies. The gene-silencing efficiency achieved 30% when the weight ratio of NP/siRNA was 4. In addition, during the preparation of NP/siRNA complexes, the NP and siRNA were diluted separately followed by mixing evenly to prevent the aggregation of the complexes for maintaining a high transfection efficiency.

Furthermore, it is crucial to select appropriate target genes. Target gene candidates in cancer therapy are mainly involved in oncogenes, angiogenesis and proliferation, like *PLK1*, *EphA2*, *RRM2*, *KRAS*, *PKN3* and *VEGF*, which have been tested as target genes in early-phase clinical trials for RNAi-based cancer therapies [7]. For GBM, mostly studied target genes of siRNA included oncogenes, cytokine receptors and key signaling regulators, such as *BCL2*, *SIRT2*, *GAL1*, *c-Met*, *EGFR*, *BAG3* and *PLK1* [8]. Nevertheless, most of these target genes are not cancer-specific, and may lead to side effects like chemotherapy. According to the previous studies, REST was a transcriptional repressor against neuron-specific genes in non-neuronal cells. It also acts as a tumor suppressor in non-neural tumors while as an oncogene in neural tumors [13,20]. Therefore, REST is a specific target for treatment of GBM along with the potential to repair nerve injury by promoting neuron differentiation. Here, we have analyzed the two most important factors in cancer development; proliferation and migration, after the delivery of the REST siRNA via PEI-coated Fe_3_O_4_ NP into GBM cells. The cell viability showed that the cell proliferation decreased, and the cell migration ability decreased to 50% by transwell assay. These results suggest that the delivery of REST siRNA via the NPs to GBM cells can effectively reduce proliferation and migration ability, providing a new way for gene therapy against GBM.

In summary, we demonstrated the use of PEI-coated Fe_3_O_4_ NP as a delivery system for therapeutic siRNA delivery. We found out the appropriate NP/siRNA weight ratio for the REST silencing in GBM cells, inhibiting cell proliferation and migration efficiently. These findings represent a novel potential treatment strategy for GBM.

## 4. Materials and Methods

### 4.1. Synthesis of PEI-Coated Fe_3_O_4_ NPs

The preparation of PEI-coated Fe_3_O_4_ NPs was carried out according to the previous reports [12,21,22]. Briefly, oleic acid-capped Fe_3_O_4_ NPs were synthesized and oleic acid was replaced by meso-2,3-dimercaptosuccinic acid. The meso-2,3-dimercaptosuccinic acid-capped Fe_3_O_4_ NPs were collected by magnetic separation and dissolved in deionized water. The NPs (0.175 ng/mL, 20 mL) were added into PEI solution (0.5 mg/mL, 20 mL) under stirring, sonicated for 20 min and stirred for 2 h. Ultrafiltration was applied for the removal of free PEI to get PEI-Coated Fe_3_O_4_ NPs.

### 4.2. NPs Characterization

The PEI-coated Fe_3_O_4_ NPs were characterized by transmission electronic microscopy (TEM, JEM-2100, JEOL Ltd., Tokyo, Japan) at 120 kV and the particle size was analyzed using ImageJ software (NIH, Bethesda, MD, USA) where the samples were prepared as described in literature [23]. DLS was carried out for the analysis of hydrodynamic size and zeta potential using Zetasizer 3000HS (Malvern Instruments Ltd., Worcestershire, UK). The thermogravimetric analysis was performed using thermogravimetric analyzer (TG 209 F3, NETZSCH, Shanghai, China).

### 4.3. Gel Retardation Assay

1 μg siRNA NC (5′-UCCGAACGUGUCACGUTT-3′, non-targeting siRNA, synthesized by GenePharma, Shanghai, China) was mixed with 0, 0.6, 1.2, 1.8, 2.4, 2.6 and 2.8 μg PEI-coated Fe_3_O_4_ NPs to get NPs/siRNA weight ratio of 0, 0.6, 1.2, 1.8, 2.4, 2.6 and 2.8, siRNA and NPs were diluted with Opti-MEM I Reduced Serum Medium (Gibco, Shanghai, China) to 10 μL before mixture. The final volume of each mixture was 20 μL and they were incubated at room temperature for 5 min. Electrophoresis was carried out using 2% agarose gel at 100 V for 20 min. The siRNA bands were visualized by DuRed (Fanbo, Beijing, China) and imaged using a Tanon-1600 image analysis system (Tanon, Shanghai, China). The gray value of the image was analyzed by ImageJ.

### 4.4. Cell Culture

The human GBM cell lines U-87 and U-251 were obtained from the Cell Bank of the Chinese Academy of Sciences (Shanghai, China). These two cells were incubated in Dulbecco’s Modified Eagle Medium (DMEM, HyClone, Beijing, China) culture medium, supplemented with 10% fetal bovine serum (FBS, HyClone) and 1% Penicillin streptomycin (Gibco, Shanghai, China) at 37 °C in a humidified atmosphere containing 5% CO_2_ [14].

### 4.5. Transfection of siRNA

The cells were seeded in 6-well plates and incubated as described above for 12 h before transfection. 1 μg siRNA NC, siREST (5′-GCUGCGGCUACAAUACUAATT-3′) and FAM-siRNA NC (FAM-conjunct siRNA NC for fluorescence detections) were diluted with 100 μL Opti-MEM I Reduced Serum Medium separately; 0, 2, 4, 6 and 8 μg PEI-coated Fe_3_O_4_ NPs were also diluted with 100 μL Opti-MEM I Reduced Serum Medium separately; The diluted siRNA was added to the diluted NPs respectively and incubated at room temperature for 5 min. Then the mixtures were added to the cells and cultured for the indicated time. All the siRNA duplexes were chemically synthesized by GenePharma. 6 h after transfection with FAM-siRNA NC, the cells were observed under a fluorescence microscope Observer A1 (Carl Zeiss, Oberkochen, Germany) and the transfection efficiencies were detected using a flow cytometry (BD Biosciences, Franklin Lakes, NJ, USA).

### 4.6. Prussian Blue Staining

The cells were seeded and indubated in 6-well plates. The transfection with NP/siRNA complexes was carried out and cultured for 6 h. Then, 4% paraformaldehyde was used to fix the cells for 30 min. The cells were rinsed with phosphate buffer saline (PBS) and stained with 1 mL of 2% potassium ferrocyanide in 6% hydrochloric acid (Perl reagent for Prussian blue staining) for 30 min. The cells were washed and the iron uptake was observed by a light microcopy (CKX31, Olympus, Tokyo, Japan).

### 4.7. RNA Extraction and Realtime Polymerase Chain Reaction (PCR) Assay

Total RNA was extracted from cells using RNAiso Plus (Takara, Dalian, China) and quantified using NanoDrop 2000C spectrophotometer (Thermo, Wilmington, DE, USA). The PrimeScript RT reagent Kit with gDNA Eraser purchased from Takara was used for the reverse transcription. The SYBR Premix Ex Taq II from Takara was used for the quantitative real-time PCR. PCR was performed on ABI 7500 Real-Time PCR System. The mRNA levels of genes were calculated using the 2^−ΔΔ^*^C^*^t^ method. Glyceraldehyde-3-phosphate dehydrogenase (GAPDH) was served as an internal control. The primers were chemically synthesized by AuGCT Biotech (Beijing, China). The sequences are as following (Table 1).

### 4.8. Protein Extraction and Western Blotting

Total protein samples were extracted by RIPA lysis buffer (Beyotime, Haimen, China) and quantified by BCA protein assay kit (Takara). The protein samples were loaded and separated by 10% sodium dodecyl sulfate polyacrylamide gel electrophoresis (SDS-PAGE) and transferred to PVDF membrane (Millipore, Beijing, China). The membrane was incubated in diluted primary antibodies against REST (1:1000, Santa Cruz, Dallas, TX, USA) and GAPDH (1:5000, Proteintech, Wuhan, China) overnight at 4 °C. After probing with horseradish peroxidase (HRP)-conjugated secondary antibodies (Proteintech) at room temperature for 1 h, protein bands were visualized using Clarity Western ECL Substrate (Bio-Rad, Hercules, CA, USA) on a chemiluminescence detection system (Tanon 5200, Shanghai, China). The protein bands were analyzed using ImageJ.

### 4.9. Cell Viability Assay

The cell viabilities were assessed by cell counting kit-8 (CCK-8, Dojindo, Shanghai, China) according to the manufacturer’s instructions. The cells were seeded in 96-well plates, transfected with NP/siRNA complexes and cultured for 24 h. 10 μL CCK-8 reagent was added and incubated at 37 °C for 1 h. The absorbance at 450 nm was detected using iMARK microplate reader (Bio-Rad, Hercules, CA, USA).

### 4.10. Transwell Assay

Transwell chambers with 8 μm pores (6.5 mm diameter, Corning, Tewksbury, MA, USA) were used for the transwell assays. The cells were seeded into the upper chambers in serum-free culture medium and complete medium was added into the lower chamber. After 12 h culture, the cells on the upper surface of the membrane were carefully removed using cotton swabs and the membrane with cells were fixed with methanol and stained with 4′,6-diamidino-2-phenylindole (DAPI, Sigma-Aldrich, Shanghai, China). The images were obtained using an Observer A1 microscope.

### 4.11. Statistical Analysis

The data was presented as the mean ± standard deviation (SD). One-way analysis of variance (ANOVA) followed by Tukey’s post hoc test was carried out for the statistical analyses, using GraphPad Prism 6 software (GraphPad Software, Inc., La Jolla, CA, USA). * *p* < 0.05 was considered statistically significant.

## Figures and Tables

**Table 1:** Primer sequences for real-time PCR.

Gene	Primer Sequence	Product Lengths (bp)
*REST*	Forward: 5′-CGCCCATATAAATGTGAACTTTGTC-3′ Reverse: 5′-GGCGGGTTACTTCATGTTGATTAG-3′	145
*GAPDH*	Forward: 5′-GCACCGTCAAGGCTGAGAAC-3′ Reverse: 5′-TGGTGAAGACGCCAGTGGA-3′	138
